# Towards Targeting PI3K-Dependent Regulation of Gene Expression in Brain Cancer

**DOI:** 10.3390/cancers9060060

**Published:** 2017-05-30

**Authors:** Theo Mantamadiotis

**Affiliations:** Department of Pathology, School of Biomedical Sciences, University of Melbourne, Parkville 3010, VIC, Australia; theom@unimelb.edu.au; Tel.: +61-3-8344-5861

**Keywords:** PI3K, cell signaling, transcription factors, CREB, PTEN, NFkB

## Abstract

The PI3K pathway is one of the most highly perturbed cell signaling pathways in human cancer, including the most common malignant brain tumors, gliomas, where either activating mutations of positive pathway effectors or loss/inactivation of pathway inhibitors occurs. Knowledge of the precise transcription factors modulated by PI3K in tumor cells remains elusive but there are numerous PI3K-responsive signaling factors, including kinases, which can activate many transcription factors. In the context of cancer, these transcription factors participate in the regulation of target genes expression networks to support cancer cell characteristics such as survival, proliferation, migration and differentiation. This review focuses on the role of PI3K signaling-regulated transcription in brain cancer cells from a series of recent investigations. A deeper understanding of this regulation is beginning to provide the hope of developing more sophisticated anti-cancer targeting approaches, where both upstream and downstream components of the PI3K pathway may be targeted by existing and novel drugs.

## 1. Introduction

Phosphatidylinositol 3-kinase (PI3K) signaling is activated upon nutrient and growth factor stimulation via numerous receptor-mediated mechanisms, typically involving growth factor tyrosine kinase receptors either directly [1] or, indirectly [2] via G-protein-coupled receptor-tyrosine kinase receptors. The regulatory subunit of PI3K, p85, binds to tyrosine kinase receptors and tyrosine-phosphorylated adaptor proteins, to activate PI3K [3]. The PI3K pathway is also integrated into other key cellular pathways via tyrosine kinase receptors, which can activate PI3K through the engagement of Ras and the resulting binding of Guanosine Triphosphate-ase (GTPase) to the Ras-binding domain of PI3K [4].

To execute the canonical signaling program, PI3Ks phosphorylate phosphatidylinositol 4,5-bisphosphate (PIP_2_) phosphatidylinositol to generate its phosphorylated derivative, phosphatidylinositol 3,4,5-trisphosphate (PIP_3_), generating second messengers that regulate cellular activities including survival, proliferation, differentiation and migration. Consequently, aberrant overactivation of the PI3K pathway over-stimulates numerous cellular processes associated with tumourigenesis, including proliferation, survival, adherence and migration. Both *PIK3CA*, the gene coding for the p110α catalytic subunit of PI3K, and AKT (Protein kinase-B), the major downstream target of PI3K, are over-expressed and/or over-activated in many tumors, including malignant brain cancer [5]. The protein, phosphatase and tensin homologue (PTEN), which dephosphorylates the first kinase-mediated activation step of the PI3K pathway, is a key negative regulator of PI3K activity and is commonly inactivated in cancer, including brain cancer. Therefore, PTEN functions as a tumor suppressor.

Gliomas describe the most common and deadly group of brain tumors, originating from resident brain cells or their immature precursors [6]. Tumors are characterized by diffuse infiltrative growth into the surrounding CNS. The World Health Organization (WHO) has standardized the classification of gliomas into four groups (I-IV) based on both histopathological and molecular characteristics [7], where Grade I gliomas are non-invasive low grade. Grade II gliomas are classed as low grade gliomas (LGG) exhibiting invasion into normal parenchyma, while Grades III and IV are classed as high-grade gliomas (HGG). Median overall survival for Grade I is >10 years and 5–12 years for LGG (Grade II), while the overall survival for Grade III ranges between 3–10 years and 1–3 years for Grade IV glioma, of which glioblastoma (GBM) is the most common type [7]. Analyses investigating the genomic landscape of HGGs demonstrate that these cancers are genetically heterogeneous [8]. The biological and cellular basis of HGGs has been the focus of numerous investigations but direct elucidation of the mechanisms driving HGG initiation, heterogeneity and drug resistance remain to be determined.

## 2. The PI3K Pathway in Cancer

Evidence for a pathogenic role of PI3K in human cancers was demonstrated by the identification of several somatic mutations in the *p85α* regulatory subunit of PI3K (*PIK3R1*) in several human colon and ovarian tumors and cell lines [9]. This inspired the search for mutations in other PI3K genes resulting in the identification of somatic mutations in *PIK3CA*, in a variety of human tumors [10,11]. A recently generated conditional PI3K-PTEN mutant mouse showed that *Pik3ca* mutations combined with *Pten* loss in the mouse ovary resulted in fast growing epithelial tumors [12].

### 2.1. The Pi3k Pathway in Tumor Heterogeneity

A *Pik3ca^H1047R^* mutation targeted to mouse mammary cells demonstrates that this constitutively activated mutant PI3K p110α catalytic subunit, also seen in many human cancers, drives tumor heterogeneity and the emergence of secondary oncogenic mutations [13]. This suggests that the PI3K pathway may be an important cancer driver and that its inhibition may minimize the acquisition of secondary mutations and delay the development of drug resistance. Investigations on the role of PI3K signaling in cancer initiating cell function in two independent studies demonstrates that the expression of the *Pik3ca^H1047R^* mutation in specific mammary cell types leads to the development of distinct tumor types, suggesting that aberrant PI3K signaling activates a multipotent genetic program in normal differentiated cells [14].

### 2.2. The PI3K Pathway in High-Grade Brain Cancer

The PI3K pathway is activated in most HGG brain tumors, including GBM, and the activation of the PI3K pathway is involved in low-grade to high grade tumor transition [15]. Significantly, when one considers the upstream components of the PI3K pathway and inactivation of the tumor suppressor PTEN protein phosphatase, which normally inhibits this pathway, up to 63% of HGGs/GBMs exhibit an alteration in one of the four genes, *epidermal growth factor receptor* (*EGFR), PTEN, PIK3R1* and *PIK3CA* [16]. Perhaps the most telling evidence for the role of the PI3K pathway in brain cancer, is that activation of PI3K pathway factors is associated with reduced survival in HGG/GBM patients [17] and the more aggressive and treatment resistant brain tumors [16]. 

There are several alterations seen in the *PIK3CA* gene in brain cancers, with the most common being point mutations *E545K* (34%) in exon 9 and *H1047R* (22%) in exon 20 [16,18]. *PTEN* is mutated via inactivating mutations or deletions in about 20% of HGGs [19]. Moreover, several mouse models demonstrate that *Pik3ca* mutations can induce tumourigenesis in a variety of tissues, almost always in cooperation with mutations in other genes including *Pten*, *Apc* or *Braf* [12,20,21]. Importantly, a conditional mouse model in which the *Pik3ca-H1047R* mutation and *Pten* deletion are targeted to neural stem cells develops rapidly growing aggressive glioma-like tumors [22].

#### 2.2.1. Current Treatments for Malignant Brain Cancer Are Inadequate

For HGG, temozolomide (TMZ) is the standard chemotherapeutic used. TMZ, is a lipophilic prodrug which can cross the blood brain barrier and induce GBM cell death by introducing alkyl groups into tumor cell DNA, leading to DNA double-strand breaks and cell cycle arrest and/or death [23]. However, due to intrinsic or acquired overexpression of the DNA alkyltransferase, O^6^-methylguanine-DNA methyltransferase (MGMT), in 60% of patients with HGGs, tumor cells are resistant to TMZ [24]. Thus, it is vital that new HGG targets be identified which can be targeted by other drugs.

#### 2.2.2. Promising Pre-Clinical and Clinical Trial Outcomes Using PI3K Pathway Inhibitors

Due to the hyperactivation of the PI3K pathway in many cancers, inhibition of PI3K pathway factors presents an attractive target for therapy and has been the focus of intensive research and numerous early phase clinical trials for HGG (reviewed in [25]). For example, the pan-PI3K inhibitor, BKM120, has been studied in the treatment of recurrent HGG based on encouraging preclinical experiments demonstrating effective tumor cell killing [26]. PI3K pathway inhibitors in HGG patients have so far yielded promising but short-term responses, exemplified by two of the most recently concluded clinical trials using the PI3K inhibitor PX-866 [27] or the PI3K downstream (mTOR) inhibitor, everolimus [28]. Overall, the evidence is that despite promising preclinical data, clinical success by targeting the PI3K pathway remains elusive. This is likely due to the intrinsic complexity of the PI3K signaling cascade, so the reductionist view of PI3K signaling as a mere linear cancer cell survival pathway needs to be considered in the context of multiple pathway nodes cross-talking with other signaling pathways which redirect signals to maintain vital cell functions.

### 2.3. The PI3K Pathway and Transcriptional Regulation via FOXO, NFκB and CREB

PI3K–AKT signals control several growth-regulatory transcription factors by both activating and inhibiting gene expression. The most pertinent to oncogenic regulation, are the forkhead box (FOXO) proteins, nuclear factor-κΒ (NFκB) and cAMP Response Element Binding (CREB) protein.

#### 2.3.1. FOXO

Forkhead box (FOX) proteins constitute a family of evolutionarily conserved PI3K-regulated transcription factors, which control diverse biological processes [29]. FOX proteins bind to DNA on corresponding gene promoters via a highly-conserved domain, the forkhead or winged helix domain, that characterizes the FOX family. The loss or gain of FOX function can alter cell fate and promote tumorigenesis. Members of the FOXO subclass share the characteristic of being regulated by the PI3K signaling pathway. AKT attenuates FOX protein transactivation activity by initiating nuclear export of FOXO proteins by phosphorylating FOXO at three conserved residues [30,31], thereby inhibiting the nuclear localization signal, interfering with DNA-binding and disrupting the association with the transcriptional co-activator CREB-binding protein (CBP-p300) [32], leading to inhibition of FOXO-mediated transcription and FOXO nuclear export and subsequent proteasomal-mediated degradation [33,34]. Specifically, FOXO positively regulates the expression of the cell cycle control inhibitor, p21^KIP^, which specifically inhibits cyclin D expression [35,36]. Thus PI3K-mediated FOXO inhibition leads to a downregulation of p21^KIP^ expression and subsequent cyclin D expression which in turn positively regulates the cell cycle, a characteristic feature of cancer cells exhibiting oncogenic PI3K activation. These PI3K-FOXO-dependent functions have been shown to also operate in brain tumor cell lines and contribute to cell proliferation and survival [37,38,39]. Further, FOXO are involved in a spectrum of functions associated with cancer cell biology, including apoptosis, DNA damage repair and oxidative stress [40]. FOXO-mediated inhibition of cell proliferation has led to the view that these factors have tumor suppressive roles [41], although this view may be too simplistic given what appears to be opposing FOXO function in glioma. A recent study examining FOXO3a expression in patient HGGs suggests a strong association between high expression and poor prognosis [42], while another study using two human GBM cell lines demonstrated that FOXO3a expression induces TMZ resistance [43]. These recent studies in GBM patients and cells, underscore the importance of FOXO in supporting brain cancer cell growth and survival and highlight the potential therapeutic importance of the FOXO transcription factors in HGG.

#### 2.3.2. NFκB

Rel/NFκB transcription factors are key regulators of inflammatory, cellular stress and immune responses and are also implicated in the regulation of cancer cell survival, proliferation, chemoresistance, invasion and tumor angiogenesis (reviewed in [44,45]). NFκB is negatively regulated by the inhibitor IκB, which sequesters NFκB to the cytoplasm. The activation of NFκB occurs through the action of IκB kinases (IKKs), which phosphorylate IκB and lead to IκB proteasomal-mediated degradation. NFκB then moves to the nucleus and forms a functional transcriptional heterodimer with Reticuloendotheliosis A oncogene (REL-A) (p65). Aberrant NFκB activity in cancer results from direct genetic aberration of the NFκB encoding genes or via cell signaling. AKT activates the transactivation potential of NFκB by at least three distinct mechanisms. First, AKT can phosphorylate the IKK α-subunit which enhances IKK activity [46,47]. Second, AKT indirectly stimulates IKK activity by targeting the phosphorylation of member molecules of the mitogen-activated protein kinase (MAPK) pathway, including the Cancer Osaka Thyroid oncogene (COT) and p38 [48,49]. Finally, AKT can also target the transactivation domain of REL-A, increasing NFκB activity [49,50]. This activation appears to be integrated into a positive feedback loop, where Akt activation of NFκB further stimulates Akt via down-regulation of the PI3K negative regulator, PTEN [51]. In some cancer cell types, the PI3K-dependent transcriptional activity of NFκB is required for oncogenic transformation. Disruption of NFκB nuclear localization has been demonstrated to inhibit oncogenic transformation, cell proliferation and induce apoptosis in pancreatic cancer cells [52,53].

NFκB signaling is constitutively activated or upregulated in GBM due to tumorigenic stress signals, including cytokine stimulus. Epidermal Growth Factor Receptor (EGFR) gene amplification or expression of a constitutively activated oncogenic EGFR mutant (EGFRvIII) leads to constitutive activation of downstream signaling networks, including the PI3K pathway. Studies using GBM cells expressing EGFRvIII, demonstrate that EGFR induces association between the docking protein, Grb2-associated binder 1 (Gab1) and the tyrosine phosphatase, src-homology region 2-domain phosphatase-2 (SHP-2). This protein complex is involved in linking EGFR to NFκB transcriptional activity and is dependent on PI3K signaling [54]. NFκB can also be aberrantly activated by numerous other growth factor receptor mediated signals which activate PI3K in GBM. Aside from EGF, platelet-derived growth factor (PDGF) has also been shown to activate NFκB in GBM cells [55] and both EGF and PDGF can activate NFκB via PI3K–IKK-dependent mechanisms [47,56] and PDGF-induced PI3K signaling has been demonstrated to directly phosphorylate REL-A [50].

Recent work investigating genomic alterations in a cohort of 893 GBM patient tissues, showed that deletion of NFκB inhibitor, IκBα, is associated with disease progression, tumor recurrence and overall shorter patient survival [57]. The role of NFκB signaling in GBM recurrence is consistent with its proposed role in GBM initiating cell (GICs) biology and proliferation, which is thought to be due to NFκB-dependent regulation of cancer stem cell genes, including CD44 and cyclin D1 (reviewed in [58]). Both CD44 (Indian Blood Group molecule) and cyclin-D1 are critical for GBM cancer cell biology and lie upstream or downstream of the PI3K pathway [59,60].

#### 2.3.3. CREB

CREB is a transcription factor with diverse roles in cell function and is activated by phosphorylation at a key serine residue through the action of diverse intracellular signaling cascades, including PI3K, MAPK, and cyclic adenosine 3′,5′-monophosphate (cAMP) signaling. CREB is constitutively activated in dividing neural stem/progenitor cells in neurogenic regions of both embryonic and adult zebrafish and mouse brains [61,62]. This contrasts with brain regions outside the neurogenic zones, where CREB is only transiently activated in restricted neuroanatomical regions under the control of specific stimuli. Furthermore, CREB has long been known to be one of the most important factors regulating neural plasticity, a phenomenon not only critical for higher order brain functions, such as learning and memory [63], but also postulated to be intimately associated with neural proliferation in the adult brain [64]. Conditional mouse mutant knockouts demonstrated that the CREB pathway is crucial for normal neural cell survival at various stages of brain development [65]. Recent human and transgenic mouse studies show CREB overexpression and/or activation in human myeloid leukemia [66], liver cancer [67], and lung cancer [68]. Elevated mRNA levels of CREB are seen in breast cancer tissue, and the level of CREB expression correlates with disease progression and survival [69]. In non-small-cell lung cancer, the expression levels of CREB and phosphorylated-CREB (pCREB) are elevated in tumors compared with adjacent normal tissues, and increased CREB expression is correlated with poor patient survival [70]. Human ovarian tumors also exhibit increased CREB expression, and ovarian tumor cell lines in which CREB expression is silenced display significantly reduced proliferation [71]. Increased CREB and pCREB expression is seen in bone marrow from patients with ALL (Acute Lymphoid Leukemia) and AML (Acute Myeloid Leukemia) compared to that from healthy patients [66]. Transgenic mice overexpressing CREB in immature myeloid cells exhibit enhanced myeloid progenitor cell survival, proliferation, and immortalization that promote increased myelopoiesis, through increased cyclin A1, B1, and D1 expression [66]. Activation of CREB by the PI3K-dependent kinase Akt occurs in adult mouse neuronal progenitor cells (NPCs) stimulated by multiple proliferation promoting mitogens, including basic fibroblast growth factor (FGF-2), Sonic hedgehog (Shh), and insulin-like growth factor 1 (IGF-1) [72].

CREB is required for efficient NPC cell survival [62] and constitutively active CREB mutant overexpression/activation causes neural hyperproliferation [61]. This was the first indication that CREB can directly modulate neural cell growth, and led to the hypothesis that CREB has a role in brain tumor biology. Recent findings demonstrate that siRNA knockdown of CREB in human glioma cell lines inhibits cell proliferation [59]. The mechanism by which CREB is activated in these cells involves PI3K pathway activation via pAkt. Moreover, the PI3K-CREB axis has been shown to modulate the expression of the cell cycle factors, cyclin B1, cyclin D1 and proliferating cell nuclear antigen (PCNA) [59]. Other studies have shown that the pan-PI3K inhibitor LY294002 inhibits rat neural progenitor cell proliferation via pAkt and pCREB attenuation [72]. Significantly, a recent report shows that pCREB is a direct target of PTEN [73], implying that CREB activity is regulated via upstream growth factor induced signaling pathways but also directly inactivated by PTEN; the phosphatase also opposing PI3K pathway activity.

### 2.4. Relevance of PI3K Signaling-Transcriptional Networks to Novel Therapeutic Strategies in Brain Cancer

As current treatments offer little hope for patients diagnosed with HGGs such as GBM, identifying novel PI3K-dependent transcriptional programs in brain cancer cells offers real hope of changing patient outcomes. This would represent a paradigm shift in our understanding of the action of PI3K in human cancer biology and would perhaps establish novel therapies targeting core PI3K pathway factors, including PI3K and mTOR, for which US Food and Drug Administration (FDA)-approved drugs which can cross the blood brain barrier already exist, including BKM120 and everolimus [74,75], in combination with drugs specifically targeting transcription factors and/or co-factors. Although the number of transcription factors investigated as promising cancer drug targets remains limited compared to the cell surface and cytoplasmic target molecules, there are rational reasons for considering that such factors will be effective targets, especially in combination with other non-nuclear molecule targets. Although drugs that target intracellular signaling pathways have markedly improved cancer patient outcomes, the almost inevitable emergence of therapeutic resistance in many cancers, including GBM, makes such treatments ineffective in the long-term. This is due to the rapid reconfiguration of cancer cell biochemical programs which bypass the normal signaling pathways and the emergence of drug resistance. Resistance mechanisms are enabled by the linearity and redundancy of most oncogenic signaling pathways, exemplified by receptor tyrosine kinase mediated pathway signaling through the PI3K and MAPK pathways. Combination targeting, aiming to inhibit both cytoplasmic signaling molecules and transcription factors, which sit at the hub of multiple upstream signals and which are less prone to mutation, compared to upstream signaling factors, would severely reduce the capacity for cancer cells to develop alternative pro-survival and pro-oncogenic adaptation signaling programs. Thus, targeting transcription factors more directly is proposed as a means toward more effective anti-cancer therapy, leading to longer-term remission and less chances for the development of therapy resistance (reviewed in [76]).

Therapies which include targeting transcription factors would be especially useful for difficult to treat cancers such as malignant brain cancer, since there has been little progress in developing truly effective therapies. Since PI3K represents a typical, relatively linear cytoplasmic signaling pathway, the deeper understanding of downstream transcriptional activation events in specific cancer cell types, including brain cancer cells, will provide opportunities to achieve much better patient outcomes, including longer progression-free survival and perhaps even long-term remission. Using, as examples, the PI3K pathway associated transcription factors, described in this review, one can envisage the rational development of novel brain cancer drug combinations comprising FDA-approved PI3K pathway drugs, such as everolimus and/or BKM120, which can cross the blood-brain barrier, and an ever-growing list of experimental inhibitors specifically targeting FOXO, NFκB, and CREB. It is important to note that the three transcription factors described here are just the tip of the iceberg of novel nuclear targets which will ultimately become clinically useful.

#### 2.4.1. Targeting PI3K and NFκB Signaling

Of the three transcription factors described herein, NFκB is the most amenable to pharmacological disruption. This is due to the multitude of targetable factors which regulate the pathway. Indeed by 2006, at least 750 inhibitors of NFκB had been described, including 198 synthetic compounds [77]. Although most preclinical research on developing synthetic NFκB inhibitors for clinical applications have focused on diseases fueled by chronic inflammation [78,79], there have been efforts to both develop cancer-specific NFκB inhibitors and repurpose existing inhibitors for anti-cancer therapy due to the importance of NFκB in the regulation of apoptosis and cancer stem cell maintenance. The more recent appreciation that inflammatory signaling in the tumor microenvironment is also critical in cancer further enhances the attractiveness of targeting NFκB.

NFκB inhibition using chemical compounds, including tailored small molecule inhibitors has demonstrated that several different tumor cell types rely on NFκB signaling to survive and grow. Dimethyl fumarate, an anti-inflammatory drug already used to treat multiple sclerosis and which potently inhibits NFκB signaling, effectively blocks human breast cancer cell growth in vitro and in vivo [80], while in chronic lymphocytic leukemia cells, the NFκB inhibitor, 6-Amino-4-(4-phenoxyphenethylamino)quinazoline, triggered apoptosis [81]. With respect to brain cancer, there is emerging experimental evidence that the inhibition of NFκB signaling modulates key oncogenic properties of both glioma stem-like cells (GSCs) and GBM tumor cells. SN50, a cell-permeable peptide inhibitor of NFκB, induced differentiation of human GSCs and reduced the invasive capacity in vitro. Importantly, in mouse xenograft models, SN50 caused a reduction in oncogenicity and an increased sensitivity to the standard cytotoxic treatments used in GBM, TMZ administration and exposure to radiation [82]. In another study focusing on GBM cells, the proteasome inhibitor MG132 triggered mitochondrial-dependent apoptosis by selectively inhibiting both PI3K and NFκB signaling [83]. Other NFκB inhibitors, including BAY117082, parthenolide, curcumin, and arsenic trioxide have also been reported to induce GBM cell death [84]. Since the invasiveness of GBM cells into the healthy brain parenchyma prevents complete surgical resection of tumors, contributing to relapse, some recent studies have focused on tackling this biological property of GBM cells. Several recent studies have demonstrated that optimal inhibition of GBM cell invasion can be achieved by simultaneous inhibition of both the PI3K and NFκB pathways [85].

#### 2.4.2. Targeting PI3K and CREB Signaling

One of the most exciting prospects for a novel transcription factor drug target in brain cancer, is CREB. As mentioned previously, CREB is an archetypal nervous system transcription factor with recently recognized oncogenic functions. Its oncogenic functions rely on signals transmitted via the PI3K and MAPK pathways. New data demonstrates that a novel CREB-specific inhibitor drug, 666-15, is a potent inhibitor of GBM cell line proliferation. This naphthol derivative disrupts CREB-CBP (Creb Binding Protein) protein-protein interaction and blocks CREB-mediated transcription [86]. In a breast cancer cell xenograft mouse model, tumor growth was inhibited at nanomolar 666-15 concentrations. Importantly, pharmacological inhibition of CREB using 666-15 is well-tolerated in vivo suggesting that pharmacological inhibition of CREB is a promising new anti-cancer therapeutic approach [87]. Both 666-15 and a different structurally related small molecule CREB inhibitor have also shown anti-tumor effects in a xenograft acute myeloid leukemia cell mouse model [88] and human lung cancer cells [89]. Preliminary data shows that 666-15 is a potent inhibitor of viability and proliferation across three independent human GBM cell lines: U118, LN18 and T98G [22]. Furthermore, in a novel PI3K-PTEN induced brain cancer mouse model, the deletion of CREB reduces malignancy and increases survival [22], suggesting that dual inhibition of PI3K signaling and CREB transactivation may be an effective treatment for GBM and other malignant brain cancers with aberrant PI3K signaling.

## 3. Conclusions

**Figure 1:**
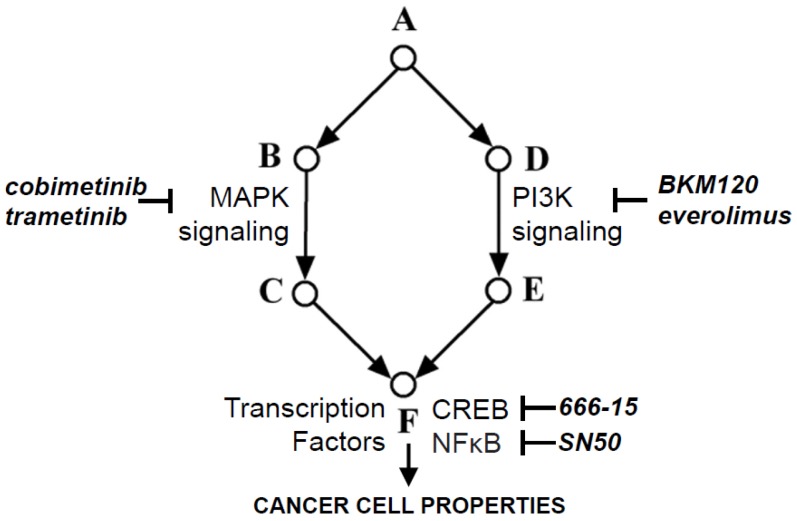
Oncogenic signaling network showing the information flow from growth factor receptors (A), via intracellular signaling pathways (B-E), where mitogen-activated protein kinase (MAPK) and phosphatidylinositol 3-kinase (PI3K) signaling are most often involved. The PI3K and MAPK pathways signal to multiple transcription factor hubs (F), including pivotal transcription factors, such as CREB and NFκB, each of which regulates the expression of hundreds of genes, the protein products of which regulate key cancer cell oncogenic properties, including survival, proliferation, differentiation, migration and drug resistance. For each targetable factor, the corresponding drug inhibitor is shown.

To achieve long-lasting, more effective management of malignant brain tumors, especially the difficult to treat forms, such as GBM, there is a need to co-target multiple pivotal points within complex signaling networks. As discussed, combining two or more drugs targeting different signaling factors, including transcription factors that are downstream at oncogenic signaling hubs should achieve more effective tumor cell stasis and/or death. For malignant brain tumors, most of which exhibit aberrant PI3K activation, targeting the PI3K pathway and one or more of its downstream transcription factors, of which NFκB and CREB are emerging as potential small molecule inhibitor targets (Figure 1), will likely lead to a paradigm shift in therapy which will benefit patients who currently have limited treatment options and only short-term benefit. It is important to note that it is highly likely that many drug combinations may have to be tested in clinical trials before the best in-class combinations are realized. Other key oncogenic pathways, such as the MAPK pathway, which is critical for cell proliferation, will also need to be targeted in many cases with existing drugs such as the MAPK small molecule inhibitors, cobimetinib and trametinib, which are being tested in the treatment of melanomas [90]. Co-targeting along multiple points within key cell signaling networks and their common transcription factor targets will minimize the bypass of cancer cell proliferation and survival mechanisms, as well as drug resistance. Lastly, expanding the array of useful drugs will arm clinicians with therapies which can be tailored to patients with distinct underlying HGG tumor biology.
